# Development and Validation the Mobile Toolbox (MTB) Spelling Test

**DOI:** 10.14283/jpad.2024.158

**Published:** 2024-09-04

**Authors:** E. LaForte, Stephanie Ruth Young, E. M. Dworak, M. A. Novack, A. J. Kaat, H. Adam, C. J. Nowinski, Z. Hosseinian, J. Slotkin, S. Amagai, M. V. Diaz, A. A. Correa, K. Alperin, M. Camacho, B. Landavazo, R. Nosheny, M. W. Weiner, R. M. Gershon

**Affiliations:** 1 Department of Medical Social Sciences, Northwestern University Feinberg School of Medicine, Chicago, IL USA; 2 Center for Health Assessment Research and Translation, University of Delaware, Newark, DE USA; 3 Helium Foot Software, Inc, Chicago, IL USA; 4 University of California San Francisco, San Francisco, CA USA; 5 Northern California Institute for Research and Education, San Francisco Veteran’s Administration Medical Center, San Francisco, CA USA; 6 625 N. Michigan Ave., 21st Floor, Chicago, IL 60611 USA

**Keywords:** mHealth apps, spelling, aging, cognitive assessment

## Abstract

**Background:**

Spelling assessments can provide a valuable marker of cognitive change in Alzheimer’s disease and related dementias (ADRD) and play an important role in ADRD research. However, most commercial assessments are not well-suited to the needs of researchers or participants; they are expensive and often require face-to-face administration by a trained examiner. To help overcome these barriers and foster progress in ADRD research, the National Institute on Aging (NIA)-funded Mobile Toolbox (MTB) offers a library of cognitive measures that can be self-administered remotely on a participant’s own smartphone, including a brand-new Spelling test.

**Objective:**

The goal of this paper is to describe the design, piloting, calibration, and validation of the MTB Spelling test.

**Design:**

We describe a pilot study, calibration study, and three validation studies, all of which use a cross-sectional design.

**Setting:**

The pilot study, calibration study, and validation studies 2 and 3 were conducted remotely, while validation study 1 was conducted in the lab.

**Participants:**

Participants for all of the studies were recruited from the general population by a thirdparty market research firm and the samples were stratified by age, gender, race, ethnicity, and education to represent the U.S. population. The pilot sample included 1,950 participants and the calibration study included 1335 participants over the age of 8. Validation study 1 included 92 participants ages 20 to 84, validation study 2 included 1021 participants ages 18 to 90, and validation study 3 included 168 participants ages 28 to 87.

**Measurements:**

Participants in each of the studies completed the MTB Spelling test. Participants in validation studies 1 and 2 completed measures from the NIH Toolbox including Oral Reading Recognition as a measure of convergent validity, and Visual Reasoning and the Rey Auditory Verbal Learning as measures of divergent validity. As an additional measure of convergent validity, participants in study 1 also completed the Spelling subtest from the Wechsler Individual Achievement Test, 4th Edition.

**Results:**

The MTB Spelling test demonstrated evidence of internal consistency (r=.79 to.83) convergent validity (r=.56 to.81, p<.01), discriminant validity (r =.23 to.36, p <.01), test-retest reliability (ICC=.63), and correlations with normal cognitive aging (r = −.06 to −.04, p >.01).

**Conclusion:**

Findings suggest the MTB Spelling test is a reliable and valid measure of English spelling abilities in general population samples, and has potential in ADRD research.

## Introduction

**A**lzheimer’s disease (AD) is most known for its memory impairments. However, some of the initial symptoms first recognized by Alois Alzheimer, the researcher and pathologist for whom AD is named, included verbal and written language impairments (1). Current research shows that language deficits can present in the early stages of AD, and worsen as the disease progresses (2, 3). Moreover, writing impairments, and spelling impairments in particular, are common symptoms of Alzheimer’s diseases and related dementias (ADRD) (4–7) and difficulties with spelling may be a particularly sensitive indicator of AD compared to difficulties with speech (8–11). As such, analysis of errors on spelling tests can provide insight into cognitive processes and help chart AD progression (12).

### Spelling Abilities and Cognitive Decline

Neils-Strunjas et al. (2006) present a schematic representation of spelling processes that explains how both typical and impaired individuals respond when confronted with a spelling dictation task. Normal English spelling processes are mediated by both lexical and phonological systems. The lexical system stores learned words as visual images; most typical spellers will rely on a lexical spelling system to spell both orthographically regular (i.e., words that are spelled like they sound) and irregular familiar words (i.e., common words in which normal letter-sound rules do not apply, such as “tough” and “knee”). Typical spellers also access a semantic-lexical spelling system, through which the appropriate spelling of a word can be selected when multiple spellings exist, provided sufficient semantic information is available. For example, a typical speller who is asked to spell the word meat may or may not produce a correct spelling without additional semantic clues; however, hearing the word used in context allows the individual to produce the correct spelling of meat instead of the homophones meet or mete. When faced with spelling an unfamiliar word, a typical speller relies on their knowledge of phoneme-grapheme correspondence, or their phonological system, to produce a plausible (but possibly incorrect) spelling.

Misspelling of orthographically irregular words tends to precede misspelling of regular words in the progression of AD, and individuals in the early stages of AD tend to spell irregular words using phonetically plausible misspellings (e.g., spelling rough as rouf) (8, 13, 14). Difficulty spelling irregular words is thought to be caused by selective impairments in the lexical spelling system, which connects the visual and auditory forms of whole words (15). There is also some evidence of impairment in phonological spelling (i.e., spelling words based on the way letters sound) for unfamiliar and nonwords (13). Finally, the working memory capacity required to temporarily store orthographic representations of words is also impaired in AD, which makes spelling longer words more difficult (16).

### Assessing Spelling with the Mobile Toolbox

Assessment of spelling is valuable both as a strong marker of crystallized intelligence (17), and to understand conditions related to cognitive change (8, 13, 14). However, most commercial neuropsychological assessments of language are not well-suited to the needs of researchers or participants; they are expensive and often require face-to-face administration by a trained examiner. To help overcome these barriers and foster progress in ADRD research, the National Institute on Aging (NIA)-funded Mobile Toolbox (MTB) offers a library of cognitive measures that can be self-administered remotely on a participant’s own smartphone.18 The MTB is offered through REDCap (19), a commonly used research platform in which researchers can (1) design smartphone-based cognitive test batteries using validated measures in the MTB library and (2) deploy and manage mobile data collection in their research studies. Spelling was developed as a self-administered mobile measure that can be used in studies of both typical and atypical aging.

In this paper, we describe MTB Spelling user interface design, the development of the test item pool, and the early research studies we conducted to assess the breadth and quality of the item pool. We also describe the results of three studies conducted to demonstrate the reliability and validity of MTB Spelling scores for measuring spelling ability across the adult lifespan.

### Proposed Uses and Interpretations of the MTB Spelling Scores

Validity evidence must be evaluated in the context of the stated purpose and interpretation of the scores.20 The MTB battery is intended as a tool for researchers to measure performance on cognitive markers in clinical and epidemiologic research studies; it is designed to be used outside the laboratory by allowing researchers to deploy tests to participants’ smartphones on a schedule determined by the specific study protocol. We sought to provide content-related validity evidence for MTB Spelling by demonstrating that: 1) the items adequately cover the important aspects of the construct of spelling in English; 2) scores rank-order individuals similarly to other established “gold-standard” spelling measures; 3) the group-level scores from the test administered in a remote, non-proctored setting are comparable to those obtained in a controlled laboratory setting; and 4) scores are sufficiently precise to reliably estimate the spelling ability of individuals on both single and repeated administrations of the test.

## Methods & Results

### Measure Development

#### MTB Measure Design

**Figure 1:**
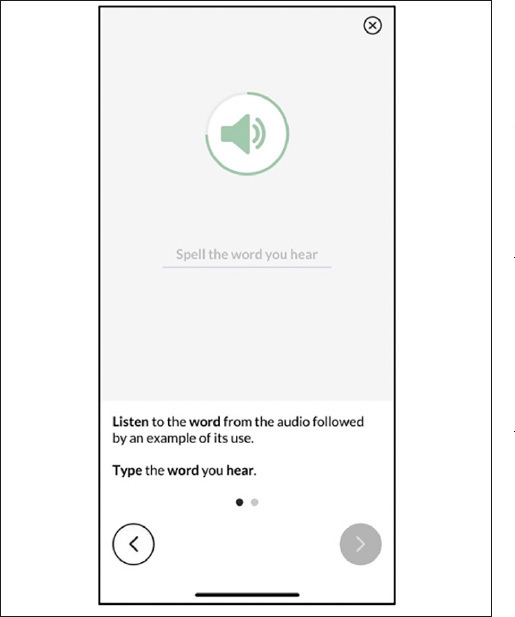
Spelling Instructions and demonstration screen (audio recording plays)

**Figure 2:**
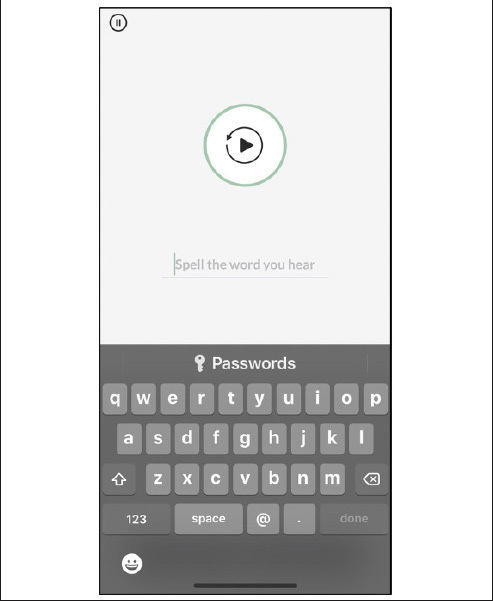
Spelling test item with keyboard activated

Like all MTB measures, Spelling starts with an instructions screen that includes an animated demonstration of the task (Figure 1), followed by one practice item. Examinees must answer the practice item correctly before the app proceeds to the test items. The Spelling items are presented via audio-recorded sound clips in traditional dictation (“spelling bee”) format: the word, followed by the word used in a short sentence, followed by the word again (e.g., Dog. My family wants to get a dog. Dog.). Immediately after the audio clip stimulus is presented, a customized on-screen keyboard becomes active, and the participant taps the keys to spell the word (Figure 2). When the participant has finished typing the word they tap the done button to submit the response and move to the next item. We intentionally omitted the autocorrect and speech-to-text features available in the native keyboard and disabled the unnecessary Shift, Number, and Space keys in the MTB Spelling keyboard. Examinees may tap the speaker icon to replay the audio prompts as needed.

MTB Spelling was designed to be a computer adaptive test (CAT). All examinees must complete a minimum of 10 items. Once the item minimum is met, the test ends when a sufficiently small standard error is obtained (SE =.44) or when the examinee has completed a maximum of 30 items. The conditional standard error implies a reliability of approximately .80 (rxx = 1 - (SE2)) which is considered generally acceptable by common standards.21

#### Item Pool Development

We reviewed lists of commonly misspelled words and selected 500 words representing all possible American English phoneme/grapheme combinations, both common and uncommon, across the range of item difficulty (Table 1). Forty-three words in the item pool have English homophone (e.g., aisle, fair, and knead). We assigned a tentative item difficulty corresponding to four grade level ranges (Table 1).

**Table 1:** Preliminary Estimates of Item Difficulty for 500 Spelling Items

**Estimated Grade Level**	**N of Words in Item Pool**	**Percentage of Words in Item Pool**
Kindergarten – 3rd Grade	15	3.0%
Grades 4th – 6th	133	26.6%
Grades 7th –12th	228	45.6%
College level	124	24.8%
Total	500	100%

### Preliminary Studies

#### Pilot Study

Sample. A sample of 1,950 individuals ages 8 through 88 (Table 2) were recruited by a third-party market research firm to complete the pilot study online. Although the task is intended for adults aged 18 years and older, we oversampled children in the pilot study to ensure adequate information about the difficulty of the easiest items in the pool.

**Table 2:** Description of the Pilot and Calibration Study Samples

	**Online Pilot Study**		**On-App Calibration Study**	
	**N**	**Percent of Sample**	**N**	**Percent of Sample**
N Ages 8 to 17	1229	63.0%	1335	67.%
N Ages 18+	721	37.0%	764	36.4%
Total N	1950	100%	2099	100%
Race				
White	1309	67.1%	1415	67.4%
Black	278	14.3%	304	14.5%
Native American or Alaska Native	21	1.1%	23	1.1%
Asian	86	4.4%	90	4.3%
Native Hawaiian or Other Pacific Islander	1	0.1%	1	0.05%
Other/Mixed	203	10.4%	211	10.1%
Not Reported	52	2.7%	55	2.6%
Ethnicity				
Not Hispanic	1657	85.0%	1786	85.1%
Hispanic	293	15.0%	313	14.9%
Mother’s Education (Ages 8 to 17)				
< High School	115	9.4%	136	10.2%
High School	471	38.3%	505	37.8%
Some College	213	17.3%	223	16.7%
Bachelor’s Degree or Higher	416	33.8%	455	34.1%
Not Reported	14	1.1%	16	1.2%
Participant Education (Ages 18+)				
< High School Diploma	25	3.5%	27	3.5%
High School	268	37.2%	294	38.5%
Some College	150	20.8%	152	19.9%
Bachelor’s Degree	226	31.3%	236	30.9%
Not Reported	52	7.2%	55	7.2%

*Participant sex was not collected in the pilot and calibration studies.

Procedure. We created 30 overlapping fixed forms of 50 items each from the 500-item pool, with each item appearing on between one and five forms. Forms were designed to have varying levels of overall difficulty based on the tentative item difficulty estimates assigned during the item pool development phase (Table 1). Each form was randomly assigned to participants based on their age and education levels (or parent education levels, for child participants).

Items on each form were presented in random order, one item per screen with a 60 second response window. Items that were presented but not completed by the examinee were treated as incorrect. The test ended when the examinee completed all 50 items or when the administration time reached 30 minutes, whichever occurred first.

Analysis. Data were calibrated using the Rasch model (22). We evaluated our fixed-form sampling design by reviewing item p-values.

Results. The number of items completed by each examinee ranged from 26 to 50 (Mean = 44 items, SD = 4.5, Median = 45 items). Approximately 3% of the examinees completed all 50 items. Two items had negative point-measure correlations; however, these items had extreme p-values (p = .01 and p = .97, respectively), suggesting that their negative point-measure correlations were likely due to the mistargeting of the items (via the fixed-form design) to the subsample of participants who encountered them. All items had acceptable mean-square infit and outfit values. Findings were used to inform the constructions of the forms in the subsequent calibration study.

#### Calibration Study

Sample. Participants ages 8 and older (Table 2) were recruited by the same market research firm as the pilot study. Participants were required to have access to a smartphone.

Procedure. Participants self-administered the Spelling test via the MTB app downloaded onto their smartphone. Items for the calibration study were distributed among 30 overlapping fixed forms of 50 items each. Each item appeared in two to four forms. Examinees were randomly assigned to one of two forms targeted to their age and education level (for adults) or parent education level (for children) with the same administration procedure as the pilot study.

Analysis. Data from the calibration study were analyzed using a 2-parameter logistic (2PL) IRT model, and the parameters from this model were used to configure the final CAT version of Spelling used in the validation studies, described below.

**Figure 3:**
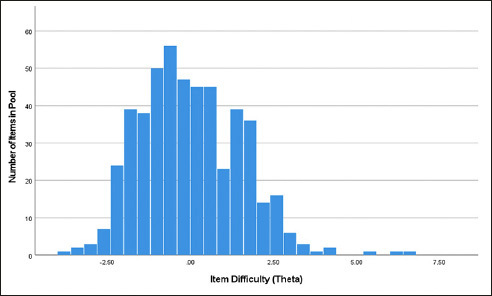
Distribution of item difficulties in the MTB Spelling item pool

Results. The number of items completed by each examinee ranged from 26 to 50 (M = 45 items, SD = 6.5, Median = 46 items). Approximately 5% of the examinees completed all 50 items. Figure 3 shows the distribution of item difficulties in the pool. Parameters from this study were used to program the final CAT Spelling measure for the validation studies.

### Validation Studies

We conducted three validation studies to understand the psychometric properties of Spelling. Participants in all three studies reflect the general English-speaking adult population (Table 3) and were not screened for cognitive or other impairments. In all three validation studies, participants took the finalized computer adaptive version of MTB Spelling via the MTB app. Due to our interest in examining monotonic relations between continuous variables, rather than strictly linear relations, Spearman Rho correlations were used instead of Pearson correlations in all correlational analyses. Across studies, we considered values of .70 or greater to be adequate for internal inconsistency (empirical and person-specific reliability) (22, 24), and used standard guidelines to judge the magnitude of Spearman rho correlations (23). All participants were unique to their respective study samples (i.e., the study samples did not overlap.

**Table 3:** Demographic Characteristics of the MTB Validation Study Samples

	**Study 1 (N = 92)**	**Study 2 (N = 1021)**	**Study 3 (N = 168)**
Missing Spelling Data % (n)	0 (0)	4.3 (44)	21.4 (36)
Age Mean (SD)	49.27 (17.65)	43.97 (21.24)	63.54 (12.10)
Age Range	[20, 84]	[18, 90]	[28, 87]
Demographic Characteristic	% (n)	% (n)	% (n)
Device Type			
iOS	100.00% (92)	63.66% (650)	100.00% (168)
Android	—	36.34% (371)	—
Gender			
Female	67.39% (62)	55.63% (568)	83.93% (141)
Male	32.61% (30)	44.37% (453)	16.07% (27)
Racial Identity			
White or Caucasian	52.17% (48)	73.65% (752)	88.69% (149)
Black or African American	32.61% (30)	13.91% (142)	4.17% (7)
Asian	9.78% (9)	6.27% (64)	2.98% (5)
Native American or Alaska Native	—	0.69% (7)	0.60% (1)
Native Hawaiian or Other Pacific Islander	1.09% (1)	0.49% (5)	—
Middle Eastern or North African	—	0.88% (9)	—
Multiracial or More Than One Race	4.35% (4)	2.15% (22)	2.98% (5)
Ethnic Identity			
Hispanic/Latino (Any Race)	1.09% (1)	14.69% (150)	7.14% (12)
Not Hispanic/Latino (Any Race)	98.91% (91)	85.31% (871)	92.86% (156)
Education Level			
Less than High School	2.17% (2)	1.67% (17)	—
High School Diploma or Equivalent	54.35% (50)	32.03% (327)	0.60% (1)
Some College	20.65% (19)	35.16% (359)	25.60% (43)
4-Year College Degree	15.22% (14)	20.27% (207)	32.74% (55)
Graduate or Professional Degree	7.61% (7)	10.87% (111)	41.07% (69)

#### Validation Study 1

The goal of Study 1 was to evaluate the internal consistency, convergent and discriminant validity, and relationship to age of MTB Spelling when completed in ideal circumstances – in a lab setting on a study-provided iPhone (which uses iOS). Study 1 data were collected in parallel with the larger NIHTB Version 3 (V3) norming study in June through September 2021.

Sample. Participants (Table 3) were recruited from a third-party market research firm who sourced participants, trained examiners to administer external assessment measures (“gold-standards”), and distributed incentives upon completion.

Procedure. Participants were first administered the NIH Toolbox® (NIHTB) norming battery by trained examiners in the laboratory. Relevant tasks included Oral Reading Recognition (a measure of reading decoding and crystallized intelligence), Visual Reasoning (a measure of nonverbal fluid reasoning skills), and the Rey Auditory Verbal Learning (RAVLT; a measure of verbal memory and learning). Participants then self-administered the MTB battery, which included the Spelling measure, on a study-provided iPhone. Finally, each participant was administered the Spelling subtest from the Wechsler Individual Achievement Test, 4th Edition (WIAT-4).25 The WIAT-4 Spelling subtest is a measure of spelling dictation accuracy in written expression, in which the examiner reads the dictation prompts, and the examinee writes responses in a paper response booklet.

Analyses. Because Spelling is a CAT, internal consistency was evaluated using average person-specific standard error-based reliability to estimate the overall empirical reliability ratio within each sample.26 We expected the mean person-specific and empirical reliability to be broadly consistent with the person-specific reliability stopping point for the CAT (SE = 0.44).

Convergent validity was examined through Spearman Rho correlations with measures of similar constructs, the NIHTB Oral Reading Recognition and WIAT-4 Spelling test. Likewise, we examined discriminant validity through Spearman Rho correlations with measures of distinct constructs, NIHTB Visual Reasoning Test and RAVLT. We also examined Spearman Rho correlations with age. Given the small education level subgroups (e.g., only 2 participants in the less than high school group), correlations with education were not examined in this sample.

Results. The correlation between the MTB Spelling theta scores and the WIAT-4 Spelling subtest scores was strong (Table 4), suggesting that the two tests rank-order examinees in a similar manner. The correlation between MTB Spelling and the Oral Reading Recognition test from the NIHTB V3 norming battery was moderate, while correlations with the fluid reasoning and memory measures from the NIHTB V3 norming battery were positive but weak. The empirical and person-specific reliability coefficients for Study 1 were above acceptable cutoffs.

**Table 4:** Results from the MTB Spelling Validation Studies

	**Study 1**		**Study 2**		**Study 3**	
	**Value**	**N**	**Value**	**N**	**Value**	**N**
MTB Spelling Theta Score Mean (SD)						
Total Sample	—	—	0.24 (0.72)	977	—	—
iOS Time 1 Theta	0.05 (0.81)	92	0.24 (0.71)	627	0.71 (0.73)	132
iOS Time 2 Theta	—	—	—	—	0.65 (0.76)	132
Android	—	—	0.24 (0.75)	350	—	—
Empirical Reliability						
Total Sample	—	—	0.79	977	—	—
iOS	0.83	92	0.78	627		—
Android	—	—	0.8	350	—	—
Person-Specific Reliability						
Total Sample	—	—	0.86	977	—	—
iOS	0.86	92	0.86	627		—
Android	—	—	0.86	350	—	—
Test-Retest Reliability	—	—	—	—	0.63	132
Correlations						
Age	−.06	92	−.04	977		—
Education			.27**	977		—
External Battery (Convergent)						
WIAT-4 Spelling Subtest	.81**	69	—	—	—	—
NIHTB V3 Oral Reading Recognition	.67**	92	.56**	968	—	—
External Battery (Divergent)						
NIHTB V3 Visual Reasoning	.36**	92	.25**	967	—	—
NIHTB V3 RAVLT Immediate	.33**	92	.23**	961	—	—
NIHTB V3 RAVLT Delayed	.22	83	.14**	905	—	—

Notes: *p < .01; **p < .001

#### Validation Study 2

The goals of Study 2 were: 1) to evaluate the reliability of the MTB scores when the tests are completed independently outside of the laboratory setting; 2) to evaluate whether there are group differences in mean MTB Spelling scores or reliability estimates by smartphone operating system (i.e., iOS vs Android device); and 3) to evaluate the strength of the relationship of the MTB Spelling scores and the NIHTB measures, when MTB Spelling is administered remotely and the NIHTB measures are administered in-laboratory. Study 2 was also conducted concurrently with the larger NIHTB V3 norming study in June through September 2021.

Sample. The recruitment and testing were conducted by the same third-party market research firm as Study 1. Participants (Table 3) were also required to have access to an iOS or Android smartphone.

Procedure. Participants self-administered the entire MTB battery, including the Spelling measure, remotely on their smartphones, within two weeks after being administered the NIHTB norming battery in the laboratory.

Analyses. As described in Study 1, in this sample we also examined reliability through empirical and mean person-specific reliability statistics. Additionally, we compared coefficients between Study 2 and Study 1 samples to understand if reliability of MTB Spelling scores is negatively impacted when the test is administered in remote, non-laboratory settings.

We examined convergent validity using Spearman Rho correlations with scores on NIHTB Oral Reading Recognition, and discriminant validity with the NIHTB RAVLT immediate and delayed scores and the Visual Reasoning Test. We also examined Spearman Rho correlations with age and education level. To determine whether there were differences by operating system, we examined the mean MTB Spelling test scores from the groups of participants using iOS and Android devices in the Study 2 sample. Mean differences were examined using a Welsch two-sample t-test.

Because all Study 2 participants were administered the NIHTB V3 norming battery in the laboratory, the sample from Study 2 provides an additional set of correlations between the NIHTB measures and MTB Spelling scores for comparison to Study 1.

Results. The empirical and person-specific reliabilities, as well as the respective reliabilities in the iOS and Android samples, were above acceptable cutoffs and similar across operating systems (Table 4). There were no significant differences in performance across operating systems (t (687) = −.01; p = .99). The correlation between scores from MTB Spelling and the NIHTB Oral Reading Recognition test was moderate, while the correlations between MTB Spelling and the NIHTB Visual Reasoning, RAVLT Immediate and Delayed tests were positive but weak. A comparison of the correlations between MTB Spelling and the NIHTB V3.0 norming tests from Study 1 and Study 2 reveal no significant differences (Oral Reading Recognition: z = 1.55, p = 0.12; Visual Reasoning: z = 1.07, p = 0.28; RAVLT Immediate: z = 1.02, p = 0.31; and RAVLT Delayed: z = 0.67, p = 0.50).

#### Validation Study 3

Study 3 was a sub-study of a larger independent validation conducted by the Brain Health Registry (BHR), an online database that facilitates aging research (27). The goal of Study 3 was to examine the test-retest reliability of MTB Spelling when taken remotely on a personal smartphone (iOS only) twice with a time delay. Study 3 data were collected from February to July 2022.

Sample. Study 3 participants were recruited from the UCSF Brain Health Registry (28) and were required to have previously opted-in to learn about research opportunities within BHR. Participants were invited by email, screened for eligibility (English fluency and access to a compatible smartphone), and provided online instructions for MTB app download.

Procedure. Participants completed MTB assessments at baseline and then again at a short-term longitudinal timepoint (approximately two weeks after baseline). We used data from the subsample of participants who completed the measure after a two-week delay, though a larger sample of participants completed the measure after one- and three-week delays as part of a separate BHR study.

Analyses. To evaluate test-retest reliability for the two-week delay sample, we used intraclass correlations from a mixed-effects model, which compared the variance in the random intercept to the total variance. Intraclass correlation (ICC) values of .60 or greater are considered adequate for test-retest reliability (29). We also compared the least squares to the expected mean differences (the statistical equivalent to a paired t-test) between groups at baseline and at the two-week follow-up.

Result. The correlation between the Time 1 and Time 2 scores was moderate (ICC = .63, n = 132; 95% CI: [.52, .73]). Participants scored slightly lower at time 2 than at time 1, but this effect was not significant (−0.14 logits; 95% CI: [−0.28, 0]).

## Discussion

MTB Spelling contains an item pool of 500 regular and irregular English words representing all 44 English phonemes and ranging in difficulty from easy (appropriate for elementary –school-aged children) to difficult (appropriate for highly educated adults). Through extensive pilot testing of the items with both children and adults, we ensured that all items in the pool exhibited acceptable model fit and discrimination statistics.

Following the pilot and calibration studies, we examined psychometric properties of MTB Spelling in three distinct validation studies. We examined the convergent and discriminant validity of MTB Spelling in Studies 1 and 2. As expected, MTB Spelling scores from our Study 1 sample were highly correlated with the gold-standard WIAT-4 Spelling subtest scores; these strong correlations suggest MTB Spelling is a valid measure of English spelling ability for adult examinees. The moderate correlations we found between the MTB Spelling scores and the NIHTB V3 Oral Reading test scores in both Study 1 and Study 2 were also expected, given that the two tests measure similar but distinct aspects of crystallized knowledge. Conversely, when we compared MTB Spelling scores to scores from tests of fluid reasoning and memory in Validation Studies 1 and 2, we observed low correlations, which supports the discriminant validity of the measure.

One useful feature of the MTB battery is the flexibility for researchers to assign participants to take assessments outside of the laboratory, on their own mobile devices (iOS and Android). The data we gathered in Study 2 showed no mean score difference between the groups of participants who took MTB Spelling on an iOS vs.an Android device, suggesting that the score interpretations are not impacted by the participant’s device operating system. Additionally, the similarity of correlations between the MTB Spelling scores and other cognitive measures for the in-laboratory Study 1 sample and the remote Study 2 sample provides additional evidence that the overall group-level test scores are not significantly impacted by administration conditions.

We also examined the reliability of MTB Spelling to ensure scores are sufficiently precise for accurately estimating individuals’ spelling ability. We examined both the precision of the test scores from a single administration of the test (using both empirical and person-specific reliability estimates) and consistency of the test scores across repeated administrations. Both the empirical and person-specific reliability indices were stable and high across all three samples. The data from Study 2 revealed that reliability from single administrations was not impacted by the type of device the test-taker was using; reliability indices were consistently high across both the iOS and Android samples of participants. The data from Study 3 showed adequate test-retest reliability of MTB Spelling scores at a 2-week time interval, and practice effects were not significant.

In our general population samples, MTB Spelling did not demonstrate a significant correlation with aging, as we would expect with crystalized ability trajectories in normal cognitive aging (30). This finding is promising from an ADRD research perspective—it is possible that MTB Spelling could differentiate healthy individuals from those with cognitive decline. However, more research is necessary in clinical samples to test this hypothesis.

### Limitations

Although the measure has demonstrated many strengths, MTB Spelling has some limitations. It is important to note that MTB Spelling is designed as a research measure and is not intended for clinical use or individual decision-making. Additionally, as the measure is intended for remote, unsupervised settings, there is the risk of a lack of attention, which would result in lower-than-expected scores, or cheating, which may result in higher-than-expected scores. Nevertheless, the convergent results between our in-lab sample (Study 1) and fully remote sample (Study 2) provide some assurance against these concerns.

Although our validation samples were robust, they are not without their limitations as well. Overall, the samples, especially from Studies 1 and 2, were quite diverse in age, race, ethnicity and education. However, representation of racial/ethnic identities aside from White/Caucasian, Black/African American, and Asian or Pacific Islander was low or missing in Studies 1 and 3, and relatively low across all samples. As such, future work is needed to establish that results generalize to these populations. Similarly, there were relatively few individuals with less than a high school diploma in our samples. Researchers should use caution when using MTB Spelling with the populations that were underrepresented in our samples until further research is conducted. Finally, although it demonstrates promise in detecting age-related changes, MTB Spelling has yet to be validated in clinical populations, and its ability to predict AD remains unclear. Studies in clinical populations and longitudinal samples are necessary to better understand the role of MTB Spelling in ADRD studies.

## Conclusion

The NIA-funded Mobile Toolbox provides a library of cognitive measures that can be self-administered on participants’ smartphone devices in a remote, unsupervised setting. Findings from three validation studies of MTB Spelling provide evidence to support the use of the test scores for group-level research. The relative cost-effectiveness and convenience of MTB Spelling make it a promising option for researchers who are interested in studying spelling impairment associated with Alzheimer’s disease and age-related cognitive changes, as well as crystallized intelligence in both clinical and healthy samples.

## Notes

*Ethical standards:* Approval from an institutional review board was obtained for each of the studies described in this paper.
